# Overview of Neurological Mechanism of Pain Profile Used for Animal “Pain-Like” Behavioral Study with Proposed Analgesic Pathways

**DOI:** 10.3390/ijms21124355

**Published:** 2020-06-19

**Authors:** Mun Fei Yam, Yean Chun Loh, Chuan Wei Oo, Rusliza Basir

**Affiliations:** 1 Department of Anatomy, Faculty of Medicine and Health Sciences, Universiti Putra Malaysia, Serdang 43400, Malaysia; 2 Department of Pharmacology, School of Pharmaceutical Sciences, Universiti Sains Malaysia, Minden 11800, Malaysia; yammunfei@usm.my; 3 Department of Organic Chemistry, School of Chemical Sciences, Universiti Sains Malaysia, Minden 11800, Malaysia; oocw@usm.my

**Keywords:** nociceptive, neuropathic, inflammatory, behavioral study, phasic pain, tonic and viscera pain, analgesic profile

## Abstract

Pain is the most common sensation installed in us naturally which plays a vital role in defending us against severe harm. This neurological mechanism pathway has been one of the most complex and comprehensive topics but there has never been an elaborate justification of the types of analgesics that used to reduce the pain sensation through which specific pathways. Of course, there have been some answers to curbing of pain which is a lifesaver in numerous situations—chronic and acute pain conditions alike. This has been explored by scientists using pain-like behavioral study methodologies in non-anesthetized animals since decades ago to characterize the analgesic profile such as centrally or peripherally acting drugs and allowing for the development of analgesics. However, widely the methodology is being practiced such as the tail flick/Hargreaves test and Von Frey/Randall–Selitto tests which are stimulus-evoked nociception studies, and there has rarely been a complete review of all these methodologies, their benefits and its downside coupled with the mechanism of the action that is involved. Thus, this review solely focused on the complete protocol that is being adapted in each behavioral study methods induced by different phlogogenic agents, the different assessment methods used for phasic, tonic and inflammatory pain studies and the proposed mechanism of action underlying each behavioral study methodology for analgesic drug profiling. It is our belief that this review could significantly provide a concise idea and improve our scientists’ understanding towards pain management in future research.

## 1. Introduction

Pain is an easily recognized sensation that is experienced by humans and animals alike. However, the process behind the production of the pain experience is a complex pathway that requires parallel integration of both the emotional and sensory experiences together with noxious perceptual information registered by multiple layers of our brain structure with the purpose of defending our body from harm’s way [1]. It has been hypothesized since the 19th century that explorers in the subject of pain have identified that similar brain structures were involved in both the production of pain perception and the process of nociception as well as its associated behavioral expression [2,3]. Unlike the polymorphic nature of pain that is often described as a sensation for us human beings, the perception of pain that occurs in animals can be examined thoroughly through their reactions in a conscious state, so to say such as in “pain-like” behavioral studies. The pain behavioral studies on animals are crucial to aid in defining and understanding the complete mechanism that is involved in the production of the pain perception and, thus, is essential in the development of new pharmacotherapy managements for overcoming the currently available analgesic which has become insufficient or inefficient in its role as a pain management drug due to its dose-limiting adverse effects [4].

Briefly, there are three main events will occur in the pain mechanism due to noxious stimulation which includes transduction, transmission and modulation of the signals. These signals will be conducted in two ways, where the upward carrying sensory information from the body to the brain via the spinal cord is known as ascending, and the signals sent from the brain to the reflex organs through the spinal cord is defined as descending pathway. Primarily, both the peripheral nervous system (PNS) and central nervous system (CNS) are involved in all types of pain perception. PNS composed of ganglia and nerves that located outside the brain and spinal cord, playing vital roles in connecting the CNS to our limbs and organs. Whereas the CNS that comprises of the spinal cord and brain is mainly functioning in integrating and interpreting the signals sent from the PNS, then immediately coordinating all the activities in our body [5]. Here, the analgesics that refer to agents that are used to relieve pain will act through the CNS or PNS mechanism pathway without significantly affecting consciousness. Analgesics can be narcotic or non-narcotic. Narcotic means that the analgesics that act through CNS but do not produce an anti-inflammatory response, such as tramadol and morphine, whereas non-narcotic will act peripherally whilst producing an anti-inflammatory effect such as non-steroidal anti-inflammatory agents (NSAIDs) [6].

In experimental designs, the pain behavioral studies on animals focuses on two fundamental components: the type of stimulus introduced to the animals (input) and the reaction portrayed by the animals (output). The aim of this study is to identify and have a complete understanding of the reactivity towards pain via the different sensory pathways of the animals; thus, the stimuli provided stem from numerous sources via the biological components of the test animals. Hence, there are multiple behavioral study methods and designs that are carried out during the experimental phase. The design of the study does not overlook the categorization of pain—phasic (thermal, mechanical and electrical), tonic and visceral, and inflammatory pain. The index of pain is quantified via the escape behavior, withdrawal reflexes, licking behaviors and vocalization of these animals. The measured variables are inclusive of the unpleasantness scores, pain intensity, amplitude of stimulation, latency of response and measurement of tolerance.

**Figure 1:**
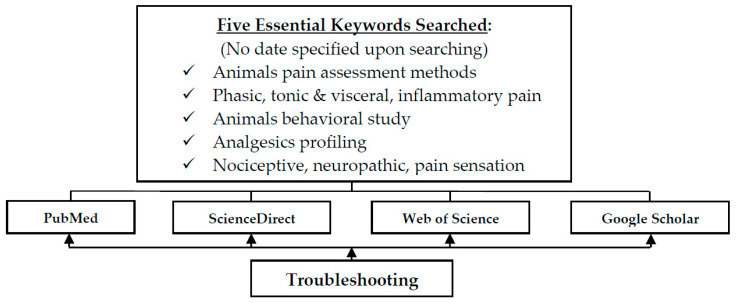
The literatures searching strategy of the present review.

There are numerous animal pain study methodologies that have been recommended and proposed in several studies; however, what is lacking has been the study of the fundamentals, the methodology to study the mechanism of actions that lead to pain perception. This part is the cornerstone in characterizing the analgesic profiles of newly discovered or developed analgesic compounds [7,8,9]. Therefore, this present review aims to summarize and propose the mechanism of actions that are involved in each demonstrated method of the animal behavioral pain study, whilst providing their respective pros and cons as shown in Table 1 and Table 2. Thus, researchers from now on would be able to effectively select the most appropriate animal behavioral study method to evaluate the mechanisms underlying as well as characterize their potential analgesic drug profile instead of conducting all the tests without a defined purpose. The flow chart (Figure 1) below is the literature searching strategy of present review.

## 2. Non-Inflammatory Behavioral Pain Study in Animal Models

### 2.1. Phasic Pain

Phasic pain is defined as a short duration and reflects the immediate impact of the onset of injury [62]. The phasic pain receptors rapidly adapt to a stimulus and have a rapidly diminishing response. Phasic pain in animals is frequently being studied using thermal, mechanical and/or electrical stimuli. The thermal stimulation will be used to evaluate Aδ and polymodal C-fiber nociceptors, whereas the chemical stimulation normally used to assess the roles of C-fibers and free nerve endings, and the mechanical stimulation will be used to assess the tactile reactivity of both C and A nerve fibers. When the stimulus is being introduced, the animals will respond via their withdrawal reflex, vocalization, paw licking or other escape behaviors, and the stimulus will then be terminated once a response is being detected.

#### 2.1.1. Thermal Stimuli

i. Tail-Flick Test

Generally, there are two variants of the tail-flick test that are tested on the tails of rats and mice; the first variant is the radiant heat tail-flick test where the rodent’s body is wrapped with a cloth with its tail exposed to radiant heat from the lamp at a specified spot on a tail-flick analgesic meter [63]. The rodent will withdraw its tail once the level of heat reaches a point of discomfort, hence terminating the heat stimulation automatically. The tail-flick latency which refers to the time taken for the animal to respond towards the heat stimulus (in a range of 46–55 °C) applied will be subsequently obtained [4,64,65]. The test will be repeated for a minimum of three times to obtain the mean value of the tail-flick latency. The major concern for this form of study is that the tail of the rodent should not expose to the heat for more than 20 s to avoid burns. The pros of this test discussed are its simplicity, non-tactile stimulus and the low inter-animal variability during the measurement. Whereas the downside to it, as mentioned in previous publications, included the prone of habituation which will cause a decreased rate of responses due to repetitive heat stimulation [66,67].

The second version of the tail-flick test would be tail immersion to either dip the tail of the animal into a cold or hot water bath. Typically, the animal will be held firmly beside the water bath and approximately half of its tail will be immersed into the hot water at 48–55 °C [68,69]. During the tail immersion study, the tail-flick latency will refer to the period of time till an abrupt movement of the tail is demonstrated or the action of the rodent recoiling its body as a reaction towards the heat stimulus applied. In this test, a cutoff system is applied to avoid damaging of the tail. By using the tail immersion tail-flick test, the area of the animal’s tail which will be in contact with the heat stimulus is greater as compared to the radiant heat tail-flick method. The major advantage of the tail immersion tail-flick test is that the temperature of the water bath can be controlled to the desired temperature, while the disadvantage is that this behavioral study is highly dependent on the animal handling capabilities of the experimenter [8]. The tail-flick methods are very effective and recommended to be utilized for studying the opioidergic analgesic profile (not opioid partial agonists) where the pain threshold can be observed to increase within 30 min post-injection of either morphine or tramadol in the subject of study [70,71]. However, both these methods are not sensitive to the analgesic effects of non-steroidal anti-inflammatory agents [72,73]. It has been used to study the putative genetic differences among animals without the drug.

ii. Hot-Plate Test

Prior to the commencement of this test, the rodents (mice and rats) are brought to the testing room for acclimatization of at least 15 min [74]. After which, the animals will be placed in an open-ended cylindrical space which is placed on top of a metallic plate connected to a thermode. The temperature is measured by a built-in digital thermometer and the temperature will be increased consistently up to a maximum of 65 °C. The animal will respond to the thermal stimulus by licking or flicking its hind paw or jumping upward (frequently at 55 °C) due to supra-spinally integrated responses. The latency of response will be recorded using a stopwatch. If the animal does not respond within 30 s (depends on type of opioids study) of introduction of heat stimulus, they are removed immediately from the hot plate. The hyperalgesic profile of each rodent can only be tested once. The mainframe of advantage for this test focuses on the accuracy and precision assurance. Measuring the reaction time of the first evoked behavior regardless of whether the subject responds by jumping or paw licking accurately provides a better insight towards the hyperalgesic profile of the subjects. However, some of the responses from the subjects are harder to be observed than others due to their relatively stereotypical behavior especially in rodents like rats and mouse where they would potentially licking their paws or trying to escape the space they are being placed in, regardless whether a heat stimulus is being introduced or not [8,75]. Besides, the hot-plate test is prone to development of a learning phenomenon for the naïve animals, which means that the reaction time will be progressively diminished and the genuine licking behavior as a response of the animal towards the heat stimuli will disappear. Besides, if the animals are habituated inside the testing chambers without noxious stimuli, the escape behavior will diminished significantly; in this case, the pain-induced jumping can be more accurately measured. Sometimes, the animals will tend to demonstrate behavior similar to when they are reacting to a stimulus even on the unheated plate (constant noxious temperature applied) [76,77,78]. 

Furthermore, this test is more recommended to study centrally acting drug analgesic profiling such as opiates [79,80], and not recommended to be used for peripherally acting drugs analgesic profiling study because the paw-licking behavior is the only behavior affected by opioids, and the jumping reaction time is increased equally by less powerful analgesics [75]. Thus, the conclusion is that the hot plate test is a delicate test to be executed for the profiling of analgesic drugs.

iii. Paw Withdrawal/Hargreaves Test

This method is recommended for measuring the cutaneous hyperalgesia due to the thermal stimulation [81]. Typically, a subcutaneous injection of λ carrageenan (also other algogens) is administered to the hind paws of mice or rats that lead to the inflammation (thermal hyperalgesia) for the execution of this study. The paw inflammation can also be induced by exposure to ultraviolet rays [82]. After which, the subject is being placed inside an enclosed glass box with a focused infrared source (~48 units) moving underneath the subject [83]. When the subject remains static, a button will be pressed which applies radiant heat (35–70 °C at intervals of 2.5 °C increasing for every 10 s) to the plantar surface of the animal’s paw [84]. Once the animal moves its paw, the photosensor will halt the time on the counting machine, and the latency is recorded. In order to prevent sensitization response of the rodents, the recommendation is for these subjects to each been studied for four sequential trials at approximately 5 min intervals between each of the test sessions. The main advantage of this test is the removal of the unnecessary retraining of the subjects; thus, both front and hind paws can be tested simultaneously, and the plantar surface is sensitive sensory skin which mimics to the skin surface of a mammalian. Besides, the Hargreaves test allows the measurement of ipsilateral and contralateral paw withdrawal temperatures, which is advantageous in unilateral pain models such as the inflammation induced by carrageenan and capsaicin that led to hypersensitivity [85]. Despite the advantages that can be observed, the cons of this test clearly demonstrate that the leg position could be a factor due to the background level of activity in the flexors that varies according to the animals’ position [86,87]; additionally, the paw withdrawal time is recorded instead of a direct measurement of the temperature [4]. Similar to the other thermal probe test, the Hargreaves test is sensitive to detect opioid-mediated analgesia, such as oxycodone and also used to discern a peripherally mediated response [85,88].

iv. Cold Stimuli

This test is less likely to be used for acute pain testing but is commonly practiced for the cold allodynia test in animals with neuropathies. The rats or mice will be placed in a Plexiglas box which is located on top of a cold plate that is cooled by the circulating cold water under it with the temperature set in the range of −5–15 °C [89]. During the commencement of this particular experiment, the room temperature should be maintained at 21 ± 1 °C. The subject will be observed to respond by brisk lifting or stamping of its ipsilateral hind paw. When the subject is observed to have such a reaction, the latency for cold pain withdrawal of these subjects will be recorded. The maximum cutoff time will be capped at 150 s in each set of the experiment. Each animal will be tested only once a day to avoid the possibility of tissue damage due to prolonged exposure to the cold surface. The major advantage of execution of a cold stimuli assay over the conventional small cylindrical hot plate testis that the test can be executed without any animal restraint, hence allowing the spontaneous behavioral study within a minimal stress condition and the temperature can be controlled accurately [4]. However, the position of the animal’s paws may vary greatly due to the animal being restrain-free [90,91].

Besides the methodology described, the cold stimuli test can also be conducted via application of acetone (100 µL) on the plantar skin of the animal [92]. The rodent will be placed on a metal mesh floor, whilst acetone begins to be vaporized, hence producing a distinct cooling sensation. The animal will respond by either biting, licking or flinching of its limb which will be taken as the response latency. Rodents with existing neuropathic pain will give an exaggerated feedback response while rodents without neuropathic pain will have little to no response towards the same stimuli [8]. This method is sensitive to assess the analgesics of opioid agonist, but not sensitive to chloropromazine, aspirin, acetaminophen, diazepam and amitriptyline [93,94]. 

#### 2.1.2. Mechanical Stimuli

i. Randall–SelittoTest

This method, also known as paw pressure test, was mainly used to assess the response thresholds to mechanical pressure stimulation, such as mechanical hyperalgesia [4,95]. Nociceptive mechanical stimuli will be applied on either the tail or the hind paw of the rodent in the Randall–Selitto test. Practically, this test is preferred for rats rather than mice as heavy physical restraining is required and the mice rarely tolerate this handling [96,97]. The animals will be restrained by wrapping a towel around their abdominal trunk. A dome-shaped pusher with the blunt tip of an analgesy meter for the rodent’s paw will be used to apply pressure on the dorsal surface of either the tail or the muscle of their paws. Pressure applied will be consistently increased at a rate of 25.5 g/sec. Once the withdrawal reflex of the paw, escape reaction of its trapped limb, or a vocal reaction is being detected, the analgesic meter will record the withdrawal threshold (in gram). In this test, the cutoff load will be capped at 300 g to with similar reasoning to prevent tissue damage. In order to increase the sensitivity of the test, the animals can be trained for several consecutive days before the experiment starts [95]. Since this method requires the rodent to be restrained, both the pituitary-adrenal and sympatho-adrenomedullary axes will be activated due to the stimulus [98], also making it sensitive to NSAIDs analgesic profiling study such as indomethacin and measuring the peripheral analgesic activity [6,99].

ii. Pricking Test

This test is actually an alternative study to the Randall–Selitto test, which is used to test for the mechanical sensitivity of rodents by introducing a mechanical stimuli; a pinprick that used to assess the presence of allodynia. Basically, the animals will be gently restrained and maintained in a natural position. The rodent pincher will then be used to apply the pinprick force between two tips. Once the animal reacts, the force displayed by the rodent pincher will be recorded as the subject’s mechanical nociceptive threshold. The threshold can represent the latency of the paw lifting respond or can represented be the frequency of withdrawal by calculating the percentage of the response towards a total of ten trials of pinprick [8].

iii. Haffner’s/Tail-Pinch Method

The Haffner’s method is completed by clipping an artery clip integrated with padded jaws on the tail of the mouse with constant application of pressure [100,101,102]. The clip is left in place for 30 s, and the applied pressure will be recorded as the pain threshold once the rodent presents an attempt to dislodge the clip by biting on it [100]. The recommendation of this method would be to place the clip on the tail of the subjects. However, it can also be placed on either the ears or toes of the subjects. The concern of this method would be damage of the tissue on the rodents if there are repetitive applications of such pressure on the same location of the subject over time [103]. This method is the best option to identify the profile of analgesic drugs that acts towards the kappa opioid receptor (ethylketocyclazocine), weak narcotics (codeine and D-propoxyphene), and strong narcotics (methadone, meperidine, and morphine) [100,103,104,105]. However, this method is not the best choice when it comes to profiling NSAIDs analgesics when the pressure is applied to a normal tissue [99].

iv. Homemade Calibrated Forceps

This method is an alternative to Randall–Selitto test that used to evaluate the pain threshold induced mechanically on either mice or rats in a minimal constraint [106]. First, the animal needs to be gently restrained on a bench in order for this test to be executed. A device that is composed of a pair of blunt forceps, equipped with strain gauges that are connected to an electronic dynamometer will be used to conduct this test. An increase in force will be applied consistently between the tips of the forceps on the subject’s tail until there is a withdrawal reflex being demonstrated by the animal either via vocalization or paw withdrawal [107]. The applied force displayed on the electronic recorder once a response is shown by the rodents will be recorded as the mechanical threshold [106]. The drawback of this testing method is that sometimes difficult to precisely measure the intensity of the stimulus [6].

v. Von Frey Filament

This test is most commonly used to determine mechanical hyperalgesia and tactile allodynia related to neuropathic pain in both rats and mice [4,41,42,108]. It is a gold standard used to determine the mechanical threshold in rodent [4]. A series of eight Von Frey hairs that is made of nylon monofilaments of various thicknesses will be inserted into a holder in order to allow the experimenter to exert a defined pressure on the punctiform point of the rodent’s paw. The logarithmically incremental stiffness of the monofilament scale from 0.008 to 300 g which is equal to 0.08 to 2943 mN (typically 0.2–13.7 mN for mice and 5.9–98 mN for rats) will be used and held perpendicularly to the plantar surface of the animal’s paw for 6–8s [109]. The immediate flinching response or sharp paw withdrawal will be taken as positive responses, at which the mechanical threshold will be recorded. The major con of this test would be its non-specificity because the subject’s low-threshold mechanoreceptors are also being stimulated simultaneously when the mechanical stimuli are being introduced. Furthermore, the animals will be unrestrained, making the stimulation harder to be applied on the subjects [8,107]. This method is used for non-steroidal anti-inflammatory drugs’ characterization [105].

In mechanical stimulation models, the major concern would be that the repetition of the mechanical force on the same punctiform area on the animals will cause a diminution and the sensitivity increased on the stimulated part, which could lead to the injury of the tissues that could call into question regards to the validity of repeated tests.

#### 2.1.3. Electrical Stimuli

i. Shock-Induced Vocalization Test

In this test, an increasing level of electric current will be applied on the animal’s tail, typically mice or rats [110,111]. The animals will respond via flinching and jumping, followed by a vocalization of pain which will be detected by an ultrasonography detector [112]. It should be duly noted that the threshold for shock-induced vocalization after discharge is considerably higher than that of the stimulus-induced vocalization [113]. This method is effective for opioid analgesics such as morphine, methadone and pentazocine [114]. One of the main concerns of this test is that the appearance of the response towards the stimuli is highly dependent on the animal species tested, thus, making comparison more challenging. Besides, to reduce the possibility of animal death due to the repetitive electric current applied, the ultrasonic stimulation of the tail was suggested.

ii. Tail Shock Test/Nelson Test

The rodent can either be held using a restrainer or is completely restrain-free during the tail shock test, also known as the Nelson test. An electric shock will be introduced to the tail of the rats or mice via a clip or through percutaneous electrodes. The electrical shock applied will be increased from 0.1 to 2 mA, and the threshold will be recorded once the animal begins to vocalize their pain [115,116]. A trial run will be conducted for at least three times in order to prevent inter-subject variability in the pain threshold determination and to eliminate the animal that fails to respond to moderate shock levels before the actual study is being conducted [111,117]. The morphine-like analgesics are effective in this model [118].

iii. Tooth-Pulp Stimulation Test

In this method, rabbits were used for tooth-pulp assay [119,120]. Before this test begins, the cavity of the subject’s tooth is usually cut through the enamel and the outer dentine, with electrodes subsequently placed on the pulp surface or the inner dentine of the rodent for stimulation purposes. Apart from that, the electrodes can also be fixed in the area of the lower incisors and put on a lead under the skin which is connected to a plug that is attached at the dorsal surface of the skull. The test is normally started by introducing a low current, at which the current will be increased regularly until the response such as biting, head flicking, chewing and licking from the animal are observed and threshold recorded. The current is applied as monophasic pulses at a preset frequency and inter-stimulus intervals by utilizing a current stimulator [121].

### 2.2. Tonic and Visceral Pain

The tonic receptors are sensory receptors which adapts slowly to a stimulus, conveying a generated action potential over time. Some of these tonic receptors could be permanently activated and it is relevant in the sense that most of the clinically relevant pains are described as either tonic pain or visceral pain which arises from deep tissues. Unlike the mechanical, thermal and electrical stimuli, the visceral pain is poorly localized and could radiate over considerable distances from the site of origin. Tonic and visceral pain in animals are commonly investigated using chemical stimuli with either formalin or a writhing test. 

#### Chemical Stimuli

i. Formalin Test

This test is predominantly used to study acute and long-lasting pain induced in the paw of rats or mice and it is considered as more satisfactory model of clinical pain to screen analgesic drugs [122,123]. Formalin, which is 37% formaldehyde, will be used as the pain-inductor. There are studies that utilized chemicals like ethylene diamine tetra-acetic acid (EDTA), complete Freund’s adjuvant (CFA), capsaicin, hypertonic saline, or bee venom; however, they are less likely used as the algogens for pain study. The site of injection and the dosage of pain-inducing agent to be administered are strictly dependent on the purpose of the study. Typically, a small volume of 50 µL of 5% (*v/v*) formalin will be injected into the soft cutaneous tissue which is usually the dorsal surface of the plantar surface of the hind paw rather than the forelimb [92]. The reason behind the choice of the hind paw as the location for injecting the pain-inducing agent is to avoid the walking patterns of the animal to be affected by the presence of the fluid in the injection site. 

Formalin-provoked pain can induce three main behavioral responses—phasic flexion, tonic flexion, and licking of the injected limb. In the intraplantar injections of the formalin in the paw of mice or rats, there will be a biphasic nocifensive behavior [124,125]; the first phase of nocifensive response will occur within 5 min post-injection of formalin lasting up to 10 min. There is a quiescent phase (sensitization) following the first phase of nocifensive reaction where the subject shows relatively lesser pain responses for a period of 10 min. Then, the second phase of responses will start on the 15th minute and lasts for a rough estimation of 40 to 60 min [126]. Fundamentally, the first phase of response is related to the direct stimulation of nociceptors such as C-fibre and low-threshold mechanoreceptors including the up-regulation of substance P, while the second phase is involving central sensitization of the rodents due to the inflammatory phenomena within the dorsal horn neurons including the up-regulation of serotonin, histamine, prostaglandin and bradykinin [123,127]. The formalin test is commonly tested using the weight-score method of behavioral rating [128], which can be assessed on four scales based on the posture of the rodents (0: normal posture, 1: injected paw stays on the ground but not supporting the body, 2: withdrawal of injected paw, and 3: licking, shaking, or nibbling of injected paw) [129]. The response demonstrated by the subjects will be marked down continuously per unit of time or at regular time intervals. Once the test is completed, the animal should be administered with an analgesic drug leading to the gradual diminishing of the pain-evoked response for the next 2 h and the gradual recovery of the inflammation site of the injected area within 7 to 10 days. The formalin test is sensitive to centrally acting analgesic agents [6].

ii. Writhing Test

Generally, this test is a recommended for preliminary assessment of anti-nociceptive activity [130]. To conduct the writhing test, chemically noxious substances, typically glacial acetic acid (0.3–0.6%), magnesium sulfate, or 2-phenyl-1,4-benzoquinone will be administered into the peritoneal cavity of the rodents (frequently mice) in order to induce the activation of nociceptors followed by an expected inflammation of the visceral [122,131,132]. Acetic acid is commonly used in the writhing test to induce pain sensation due to its mechanism of action that leads to production of a localized inflammatory response from the release of free AA from tissue phospholipids via COX-producing prostaglandin [133]. The animal will be placed in a small cage for 10 min prior to the experiment and, once the algogenic agent has been injected, the writhing response should be able to be observed within minutes, indicating visceral pain. The writhing reaction such as stretching, extension of hind legs, contraction of the abdomen until touching the floor, tension to one side or twisting will usually begins 5 to 10 min post injection and lasts for about an hour [134]. The abdominal cramps per unit of time will be recorded for about 15–30 min [8,135,136]. One of the major downsides of this test is the diminishing frequency of “writhing” over time, causing the evaluation of analgesic duration in the rodents to be more challenging. However, the acetic acid-induced writhing test is recommended for testing of peripherally acting drugs (chlorpromazine, antihistamine and meprobamate) and their analgesic profile study [135,137]. 

Furthermore, the proposed mechanisms of actions on each behavioral study methodology for analgesic profile characterization evaluated in previous studies are shown in Table 1.

## 3. Inflammatory Pain Study in Animal Models

Inflammatory pain in animal models have been widely used and accepted as the study model to understand the mechanisms of tissue injury-induced persistent pain. The animal models of tissue injury and inflammatory hyperalgesia can be provoked by various inflammatory agents. Amongst them, the CFA and λ carrageenan are the most frequently used for the purpose of animal inflammatory pain studies. However, currently there are still no existing models that could potentially simulate all the symptoms of an inflammatory pain.

### 3.1. Complete Freund’s Adjuvant

Previously, the polyarthritic and persistent pain studies in animals were developed by inoculating the rodent’s tail in the *Mycobacterium butyricum* oil suspension. However, this model is not frequently being used due to the concomitant illness that typically develops in rodents following the polyarthritic induction test. Generally, CFA is a well-known, dose-dependent inflammatory agent that is normally injected into the footpad of animals to provoke a local inflammation response and a persistent pain in the subjects within minutes to hours post-injection, with the peak effect observed at about 6 to 8 h [138,139]. In rats, approximately 30–200 µg of CFA is injected in the hind paw of the animals and it can result in extreme edema within 24 h and both the peaks of hyperalgesia and allodynia can be observed within 5 h post-injection lasting for a minimum of 2 weeks [140,141]. These phenomena mimic rheumatoid arthritis occurring in humans [142,143]. From the previous studies executed, Fischer 344 (FIS) rats demonstrated a higher thermal hyperalgesia sensation in comparison to the Lewis (LEW) and Sprague Dawley (SD) subtype of rats based on the computational scoring differences by subtracting the paw withdrawal latency (PWL) of the contralateral paw from the CFA-injected paw [140]. The major disadvantage of this model study is that the rats will experience minimal weight reduction with normal grooming behavior [138].

### 3.2. Carrageenan Model

Generally, there are three main types of carrageenan available such as the iota, kappa and lambda. The lambda form is commonly used to induce acute inflammatory responses in animal models due to its gel state in room temperature [144]. The administration of this algogen was the same as described in Section 2.1.1. (iii). Typically, the intraplantar injection of 100 or 25 µL of 1% (*w*/*v*, suspension in saline) λ carrageenan to the rat or mouse paw, respectively, to cause a local inflammatory effect with two phases (biphasic) of paw edema development observed. The first phase of paw edema will occurs in the rat within the first 30 min post-injection due to the release of pro-inflammatory factors including serotonin, bradykinin, histamine and prostaglandins, whereas the second phase will begin at the end of the first hour post-injection and lasts until the third hour, with its peak being observed within 3–5h of carrageenan injection due to the attribution of neutrophil infiltration, nitric oxide and continuing prostaglandin generation [140,145,146,147]. However, the early phase of paw edema happening in the mouse will last for 6 h and followed by the second phase response that peaks at about 72h [148]. On the other hand, the injection of carrageenan will induce the formation of thermal hyperalgesia which peaks on the third day and 4th hour post-injection, respectively. Thermal hyperalgesia typically will last for almost 96 h once it is induced [138]. The cardinal signs will be resolved completely within 2–3 days which is apparently a much shorter duration as compared to the CFA-induced inflammatory model [149]. According to previous studies, the FIS rats demonstrated the highest significance in terms of thermal hyperalgesia compared to LEW and SD rats upon 3.5 mg of carrageenan administration [150]. The proposed mechanisms involved in carrageenan-induced thermal hyperalgesia and mechanical allodynia is mediated via norepinephrine and serotonergic pathways to exhibit analgesic response [59,151], whereas the anti-inflammatory action of analgesic drugs on the carrageenan-induced edema is mediated via the down-regulation of both the substance P and prostaglandin E_2_ [152].

### 3.3. Formalin Model

Refer to the subtopic of *Formalin test*.

### 3.4. Zymosan and Mustard Oil Models

Both zymosan and mustard oil can induce inflammation with hyperalgesia response via the activation of TRPV channels, which will cause excitatory effects on the primary afferent nociceptors [153]. However, the effects of both these inflammatory agents are relatively short and only can last for up to 20 min [149]. The topical application of mustard oil to the lateral surface of the rat’s hind paw can induce slight edema and plasma extravasation [154], whilst frequent biting and vocalizations can be observed from the tested subject. This phenomenon can last up to 7 min post-application. The application of mustard oil can facilitate the tail-flick test by enhancing the rate of reflex within 5 min, reaching its peak at approximately 20 min of post-application [155]. Application of zymosan intraplantarly can result in a time-dependent and persistent thermal and mechanical hyperalgesia response. Typically, the mechanical hyperalgesia will appear upon the application of more than 1.25 mg of zymosan, peaking on the 4th hour in rats [156]. Thermal hyperalgesia will occur in two phases: the early and latephase. The earlyphase peaks at 30 min post-application if ≥ 2.5 mg of zymosan is applied, whereas the latephase peaks at the 4th hour upon application of ≥0.0625 mg of zymosan. Furthermore, edemas will appear if ≥ 2.5 mg of zymosan is applied, and will reach its peak at 30 min post-administration. The duration of responses is highly dependent on the dose of zymosan applied. Additionally, the higher dosage such as ≥ 5 mg of zymosan will cause a spontaneous pain in the rodents [156]. 

### 3.5. Capsaicin Model

The application of the capsaicin will stimulate nociceptors to cause a neurogenic inflammation via activation of the TRPV1 channels. Typically, hyperalgesia, allodynia, and flare reactions will potentially appear upon the injection of capsaicin intradermally in rats (10 µg/10µL in 10% ethanol and 2-hydroxypropyl BETA cyclodextrin). For instance, the primary hyperalgesia induced by capsaicin towards a punctuate stimuli covers a larger area than those reacting to a stroking stimuli, followed by the flare response will be observed and extended to secondary hyperalgesia [157,158]. The visual flare will occur within seconds of capsaicin post-injection whereas the hyperalgesia occurs immediately upon injection, reaches its peak within 15 to 30 min and lasts for 21 h in the case of rats [159]. The same goes for the hyperalgesia reaction towards stroking stimuli, the peak will appear at about 15 min but only last for a maximum of 6 h [157]. The inflammatory effects induced by capsaicin are dose-dependent and may vary according to the area of the subject being injected. Additionally, the intraplantar injection of capsaicin in the rat’s paw can provoke neurogenic inflammation that persists for 4 h and thermal hyperalgesia that persists for 45 min maximum [149,160]. 

### 3.6. Bee Venom Model

This test was first developed by [161] to study the nociceptive effects in rats. In this test, the bee venom will be injected subcutaneously into the hind paw of the rats. Subsequently, the nocifensive behaviors such as lifting, flinching, or licking of the injected paw will be observed with it usually persisting up to 2 h, followed by thermal hyperalgesia, mechanical allodynia and edema development within 72–96h post-injection [162]. In addition to that, the thermal hyperalgesia could appear in the contralateral hind paw as well. The duration of spontaneous pain-related behavior responses are dose-dependent in both time course and response intensity [149]. This test was claimed to be sensitive to pharmacological intervention by non-steroidal anti-inflammatory drugs and morphine in demonstrating the analgesic effects [149]. The onset and duration of action of different inflammation-inducing agents were summarized in Table 2.

## 4. Assessment of Arthritis and Inflammatory Pain in Animal Models

The measurements of the inflammatory pain in animal models is strictly dependent on the behavioral changes on the animals, including the foot posture and gait analysis, dynamic and static weight bearing, thermal and mechanical sensitivity of the paw, and the spontaneous mobility [163,164,165,166]. 

### 4.1. Weight Bearing

The weight bearing test is commonly used as the main assessment on the measurement of inflammatory pain in arthritic models. Initially, the restrained animal will be placed in an angled Plexiglas chamber and the force exerted by each hind limb will be detected and measured (in grams) by the incapacitance tester over an average time of 5 s [165]. Besides that, the stepping force of each limb can also be measured by using the force sensor plates while the animal is allowed to walk through an enclosed walkway. The walking pattern of the animal across the walkway is recorded by a camera, which is fixed at the bottom of the transparent glass floor. The digitized output and the simultaneously videotaped images will be synchronized manually in order to obtain the peak vertical weight bearing by each limb. Eventually, the force exerted by each limb will be expressed as the percentage of either their body weight or the sum of the force exerted by both hind paws. The stepping force difference between each hind limb will further be calculated as a ratio for clearer comparison [167]. 

Furthermore, the stepping force exerted by each limb across the glass floor can also be evaluated by the gait analysis system, also known as “CatWalk”. A white fluorescent tube will be used as the indicator for the point of contact by the paw of the animals while the animals walk through the corridor, and the intensity of the resulting illumination will be measured. At the same time, a wide-angle CCD camera is positioned under the corridor to record the walking patterns. The major concern of this test is that the voluntary (motivation) of the animals to walk across the walkway. If the subjects refuse to walk across the path, there will be no results generated [8]. 

### 4.2. Gait and Posture Analysis

Fundamentally, the combination of gait and posture analysis is frequently used for pain-related functional impairment studies [168]. Before the experiment starts, an electrode is attached to the plantar surface of each hind paw. After that, the animal is placed on 30 cm diameter stainless steel cylinder which will be rotating at 4 rpm in order to force the animal to walk. The circuit is terminated once the electrode is in contact with the cylindrical floor, whilst the time of the circuit remained closed is recorded. The behavioral signs that could be included during this test are foot aversion, non-/partial weight bearing, toes curling, prevention of contact with the limb. Eventually, the ratio of the contact time of the control foot to the affected foot is calculated as well as the paw elevation time [8].

### 4.3. Spontaneous Mobility

This test is commonly used for knee joint arthritis pain models by studying the locomotor activity using activity boxes or the biotelemetry system. The biotelemetry system is frequently used to assess the spontaneous mobility and the temperature of the body in the animals. Initially, the transmitter of the biotelemetry system is implanted in the peritoneal cavity of the animal, and a receiver is placed under the cage. The locomotor activity of the animal will cause the transmitter to release a signal which is relayed by consolidation of matrix into the peripheral processor which is connected to a computer. Subsequently, radio waves and the activity are received as counts by the receiver [164]. Another method has been used for spontaneous mobility assessment in animals; that is, activity boxes which are made of photobeam and phototransistors. The phototransistors will be placed on the wall opposite to the photobeam, which will be activated once the animal’s movements have interrupted the beam. The photobeam patterns and the frequency emitted through the movement of animal will be subsequently recorded as an activity score [169]. 

### 4.4. Thermal and Mechanical Sensitivity of Paws

Both Randall–Selitto and Von Frey filaments tests are used to evaluate the mechanical sensitivity of the animal’s paw with knee joint arthritis. Apart from this, both the paw withdrawal and hot plate test are also used for assessing the thermal sensitivity of arthritis rodent’s paw. Refer to Randall–Selitto, Von Frey filaments, paw withdrawal, and hot-plate test mentioned above. The significantly reduction of both the withdrawal latency and withdrawal threshold of the affected limb will be observed in this test [170].

### 4.5. Mechanical Sensitivity Test

Calibrated forceps are frequently used for evaluation of the mechanical sensitivity of the arthritis knee in animals especially mouse and rats, please refer to calibrated forceps as mentioned above [171,172]. 

### 4.6. Struggle Threshold Angle 

The knee extension angle can be used to evaluate the mechanical sensitivity of arthritic knee in the study subjects. Initially, the animal is gently held upward by using one hand, and the tibia of the animal is extended to a point where the subject shows signs of struggling. The distance of the heel of the foot during the extension is measured, and the angle of the extension is calculated. Typically, the struggle threshold angle of the arthritis knee will be significantly reduced compared to the control [173]. 

### 4.7. Vocalization Threshold

In this test, the vocalization threshold is measured by compressing the arthritic knee of the restrained animals using a pair of calibrated forceps. Both the audible and ultrasonic vocalizations (approximately 2 min) are detected and measured simultaneously upon pre- and post-stimulation by using a recording chamber integrated with computerized analysis system [173,174]. The ultrasonic vocalization of the rodents represents its emotional response, whereas the audible vocalization is representation of its nocifensive response. Typically, the frequency used for the detection of audible range is 20–16 kHz, whereas the ultrasonic range is 29–21 kHz. The duration and rate of vocalization will be increased in animals suffering from knee joint arthritis [174]. Eventually, the vocalization during stimulation (VDS) and vocalization after discharge (VAD) will be interpreted individually.

### 4.8. Plethysmometer and Micrometer Measurements

Both of these devices are used to assess the edema as a surrogate of pain induced by the algogens in the paw of the rodents (rats or mice) as well as to evaluate the effectiveness of anti-edematous agents [175,176]. The measurement principle applied in Plethysmometer is based on Archimedes Law by measuring the fluid’s displacement upon the immersion of the rat’s paw in the measuring vessel, the displacement of the fluid is reflects to the exact volume of rat’s paw swelling. Typically, the fluid (mercury or water) displacement will range from 0 to 0.5 mL for mice and 0 to 7 mL for rats, whereas the micrometer is a simple device that used directly to measure the thickness of the rat’s paw edema [177]. There are different types of enhanced Plethysmometer being used by scientists to evaluate the rat’s paw edema; however, the same principle is applied. 

### 4.9. Grimace Scale Test

The grimace scale is a standardized behavioral coding system specially designed for animal pain studies that involves the noxious stimuli of moderate duration (within 10 min to 4 h) whilst accompanied with facial expressions caused by pain. The animals will be placed in Plexiglas chamber with a digital video camera (preferred high resolution) positioned at either end of the chamber. Then, the video camera will be set to record 30 min before and after the noxious stimulation applied, typically zymosan or CFA, to capture the facial expressions of the animals for grimace scoring purpose. Manually, the experimenter will screen, randomly scramble and score the face images of the animals before being told that which group of the animals has been treated. This manual image selection method was replaced by the automated frame capture using the Rodent Face Finder. After selection, each image will be given a score of 0 (not present), 1 (moderately visible) or 2 (severe) based on the facial features described in [178], then the mean grimace scores will be calculated as average score across the action units [179]. Generally, the grimace scale will quantify the facial changes of the animals (rats or mice) in a number of “action units”, and this number is directly proportional to the stimulus intensity. There are five facial features (action units) can be observed in both mice and rats including the orbital tightening, nose bulge, cheek bulge, whisker change and ear position change as described by [178]. However, there is a major exception in the cheek or nose because the bulging occurs naturally in rats, but not in mice [180]. The noxious stimuli that are applied superficially will yield a lower score compared to deep tissues such as viscera. The advantages of this test are that no preliminary animal training or special equipment are required and the measurements of the behavioral response from the animals are spontaneously emitted by themselves. In addition, the major drawbacks of this test are the labor-intensive nature of the frame grabbing in the case of manual operation and the limited duration of the pain expressions from the animals. This test is frequently used for morphine-like analgesics studies.

## 5. Limitations of Present Study

As aforementioned, there are numerous types of animal “pain-like” behavioral study methods existing so that the global scientists able to choose the testing methods that best suit their analgesics studies. Additionally, there are numerous reports showing that the researchers had purposely modified the conventional behavioral testing methods such as using different animal species and different parameters setting to run analgesics test. Apparently, these modified methods are not frequently being employed by other researchers which is resulting in limited feedbacks on these modified methods. Despite reporting the scattered information of these modified methods, we choose to elaborate the most frequently used methods as well as the type of animal species along with the benefits, drawbacks and proposed analgesics profile for each method in the present review.

## 6. Conclusions

To sum it all up, the hot plate technique and the Von Frey filament study are the two single most frequently conducted experiments to study the stimulus-evoked pain behavior including both the hyperalgesia and allodynia over time in rodents upon the injection of algesic agents. On the other hand, CFA and λ carrageenan are easily the most frequently used agents to induce chronic or acute inflammatory pain, respectively, with accompanying edema development in the test subjects. The stepping-force analgesic meter with the incorporation of a charge-coupled device camera is by far the most practical method used to investigate the variation of the stepping force of the rodents in response to the inflammatory pain induction, since the locomotion pattern of the rodents can be synchronized with the stepping force concurrently. As a matter of fact, there has not been a single behavioral assay that could capture the full spectrum of nociception in non-communicating subjects. Therefore, the pros and cons of each behavioral test method along with the proposed mechanisms preferred should be accounted for and act as a meter while developing a novel analgesic therapeutic drug in order to provide a precise and concise set of mechanism of action profile, and thus improve our understanding towards the management of pain and its evolving future.

## Figures and Tables

**Table 1:** The summary of different animal “pain-like” behavioral study methods, benefits, drawbacks and the respective proposed mechanisms of action for analgesic profile characterization.

Types of Stimuli	Test Methods	Benefits and Drawbacks (* Most Sensitive Analgesics)	Proposed Mechanismsfor Analgesia Profile Study	References
***Phasic Pain (Nocifensive Tests)***				
Thermal	1. Tail-Flick Radiant heat Tail immersion (Cold/Hot)	Pros: *Tail-Flick* Simplicity Non-tactile stimulus Low inter-animal variability during measurement *Tail immersion* Heat stimulated area is larger for tail immersion Water temperature can be controlled Cons: *Tail-Flick* Prone of habituation Possibility of over-burnt of animal’s tail *Tail immersion* Possibility of overheating the animal’s tail Highly dependent on animal handling skills (* Opioid analgesics)	-Opioidergic -Adrenergic -Serotonergic -ASICs channels -TRP channels -Purinergic -Histaminergic -Cannabinoidergic	[10,11,12,13,14,15,16,17,18,19]
	2. Hot Plate	Pros: Rapid and inexpensive test Repetitive test on same animals in short duration without causing injury on tissue Cons: Prone of habituation for naïve animals More complicated than other thermal assays (* Centrally acting drug analgesics)	-Serotonergic -Adrenergic - Na_V_, Ca_V_, K_V_ and Cl_V_ channels -Nicotinergic -Opioidergic -COX -Cannabinoidergic	[10,17,18,20,21,22,23,24]
	3. Paw Withdrawal/Hargreaves Test/Plantar Test	Pros: Animal restraint is not required to enable both front and hind paws to be tested simultaneously Ipsilateral and contralateral paw withdrawal temperatures are measurable Cons: Leg position of the animals could vary according to animal’s position Paw withdrawal time is recorded instead of direct measuring the temperature (* Opioid analgesics)	-Neurotrophin -Purinergic -Substance P -Histaminergic -Cannabinoidergic	[16,25,26,27,28,29,30,31,32,33]
Mechanical	1. Randal-Selitto	Pros: Recommended for studying neuropathic pain where both fore- and hindlimbs are affected. Cons: Animal restraint is required Preliminary animal training is required to increase the sensitivity of test (* NSAIDs and peripherally analgesics)	-Serotonergic -Cannabinoidergic -COX -ASICs channels -TRP channels -Substance P -Histaminergic -GABAergic	[16,24,34,35,36,37,38]
	2. Pricking Pain	Pros: Alternative to Randall–Selitto test Simple and rapid test Cons: Animal restraint is required to maintain the animal in natural position (* NSAIDs analgesics)	-Serotonergic -Cannabinoidergic -COX	[24,38]
	3. Haffner’s/Tail-Pinch	Pros: Simplicity and rapid test Cons: Possibility to cause injury on the site of pinch in case of repetitive test over a short course (*κ-opioid, weak and strong narcotic analgesics)	-Cannabinoidergic -COX -Opioidergic	[17,24]
	4. Homemade Calibrated forceps	Pros: Alternative to Randall–Selitto test Simplicity and inexpensive test Cons: Minimal restraining of animals is required Difficulty to precisely measure the intensity of stimulus (* NSAIDs analgesics)	-Cannabinoidergic -COX -GABAergic	[12,24,39,40]
	5. Von Frey Filament	Pros: No animal restraint is required Rapid and simple test Cons: Non-specificity due to animal’s low-threshold mechanoreceptors will also be stimulated simultaneously when the stimulus applied (* NSAIDs analgesics)	-Neurotrophin -COX -Cannabinoidergic -NA_V_ channel -TRP channels -ASICs channels -Purinergic -Substance P -CGRP -Histaminergic -GABAergic	[11,12,24,25,26,27,29,30,31,32,36,39,40,41,42,43,44,45,46,47,48,49,50,51,52]
Electrical	1. Shock-Induced Vocalization	Pros: Simple and rapid test The response is far more immediate compared to other tests Cons: The response upon stimulation is highly dependent on animal species Possibility of animal death and anxiety development due upon repetitive electric current applied in short course (* Opioid analgesics)	-Serotonergic -COX -TTX-R Na channels	[47,52]
	2. Tooth-Pulp Stimulation		-Serotonergic -TTX-R Na channels	[47,52]
	3. Tail Shock/Nelson Test		-Serotonergic -Opiodergic -Purinergic -NMDA receptor -TTX-R Na channels	[47,52,53]
***Tonic and Visceral Pain (Inflammatory Tests)***				
Chemical	1. Writhing Test	(* Peripherally acting analgesics)	-Serotonergic -Opioidergic -Peritoneal mast cells -ASICs channels -COX -GABAergic -Cannabinoidergic	[10,15,17,40,54,55,56]
	2. Formalin Test	(* Centrally acting analgesics)	*Early phase* -Serotonergic -Opioidergic -Substance P -Cannabinoidergic *Late phase* -Serotonergic -NO/cGMP/K_ATP_ pathways -Opioidergic -Histaminergic -COX -GABAergic -Cannabinoidergic	[15,17,40,56,57,58,59,60,61]

Notes: ASICs: Acid-sensing ion channels; cGMP: Cyclic guanosine monophosphate; CGRP: Calcitonin gene-related peptide; COX: cyclooxygenase; Ca_V_: Voltage-gated calcium channels; Cl_V_: Voltage-gated chloride channels; GABA: Gamma aminobutyric acid; K_ATP_: ATP-sensitive potassium channels; K_V_: Voltage-gated potassium channels; NSAIDs: Non-steroidal anti-inflammatory drugs; NO: Nitric oxide; NMDA: N-methyl-D-aspartate; Na_V_: Voltage-gated sodium channels; TRP: Transient receptor potential channels; TTX-R: tetrodotoxin-resistant.

**Table 2:** The onset and duration of action elicited by different inflammatory agents and its sensitization caused.

Inflammatory Agents	Quantity Applied	Hyperalgesia	Allodynia	Onset of Action (within)	Duration of Action (≤)
λ Carrageenan	100 µL of 1% (*w/v*)	+ (t)	+ (m)	30 min	3 days
Formalin	50 µL of 5% (*v/v*)	+	+	5 min	60 min
Complete Freund’s Adjuvant	1:1 dilution in phosphate buffered saline	+	+	5 h	2 weeks
Mustard Oil	0.0625 - ≥ 5 mg	+	+	5 min	60 min
Zymosan	D	+ (t/m)	+	30 min	24 h
Capsaicin	10 µg/10µL in 10% Etol and 2-hydroxypropyl BETA cyclodextrin	+ (t/m)	+	1 min	21 h
Venom	D	+ (t)	+ (m)	1 min	96 h

Notes: -: to; +: Presence; D: Depends on the types of agents used; Etol: ethanol; m: mechanical; t: thermal.
